# Circular RNA Expression Profiling by Microarray—A Technical and Practical Perspective

**DOI:** 10.3390/biom13040679

**Published:** 2023-04-17

**Authors:** Yanggu Shi, Jindong Shang

**Affiliations:** Arraystar Inc., 9430 Key West Avenue #128, Rockville, MD 20850, USA

**Keywords:** circular RNA, circRNA, circRNA microarray, circRNA-seq, circRNA profiling, microRNA sponge

## Abstract

Circular RNAs, as covalently circularized RNA loops, have many unique biochemical properties. Many circRNA biological functions and clinical indications are being continually discovered. Increasingly, circRNAs are being used as a new class of biomarkers, which are potentially superior to linear RNAs due to the unusual cell/tissue/disease specificities and the exonuclease-resistant stabilized circular form in the biofluids. Profiling circRNA expression has been a common step in circRNA research to provide much needed insight into circRNA biology and to facilitate rapid advances in the circRNA field. We will review circRNA microarrays as a practical and effective circRNA profiling technology for regularly equipped biological or clinical research labs, share valuable experiences, and highlight the significant findings from the profiling studies.

## 1. Introduction

**Figure 1:**
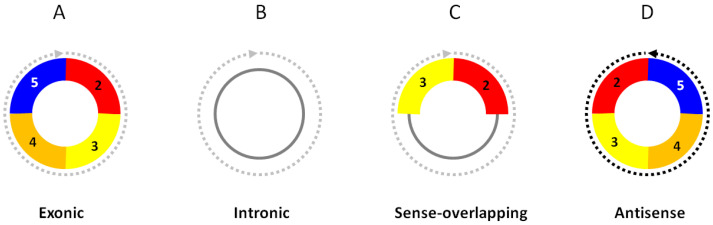
Structural categories of circRNAs. (**A**) *Exonic circRNAs* consist of only exons. (**B**) *Intronic circRNAs* consist of only introns. (**C**) *Sense-overlapping circRNAs* consist of exons and introns. (**D**) *Antisense circRNAs* are in strand direction antisense to their linear RNA counterparts. The exon numbers are for illustration only.

CircRNAs, as a new large class of RNA molecules, were initially discovered as exon-scrambled RNA transcripts in an attempt to identify possible mutant RNAs produced from genomic translocations or rearrangements in cancer cells [1]. These RNA transcripts had downstream exons that joined to the upstream exons at the characteristic “back-splice” sites, which could not be explained by the conventional linear splicing of their primary transcripts. The back-splice sites turned out to be the circular junctions of circular RNAs. Numerous circular RNAs have been discovered in all cells and tissues studied [2,3,4,5,6,7,8]. Most circRNAs are *exonic circRNAs*, which are produced from the mRNA host genes, using the same exon boundaries as their linear mRNA counterparts (Figure 1A). Some circRNAs are entirely composed of intronic sequences as *intronic circRNAs* (Figure 1B), or have both exons and introns as *sense-overlapping circRNAs* (Figure 1C). CircRNAs can also be in a strand direction that is opposite to their linear mRNAs as *antisense circRNAs* (Figure 1D) or from intergenic lncRNA genomic loci as *intergenic circRNAs*. The same exon numbers in the “best transcript”, i.e., the longest linear RNA transcript isoform from the host gene, are retained in the circRNAs by convention.

**Figure 2:**
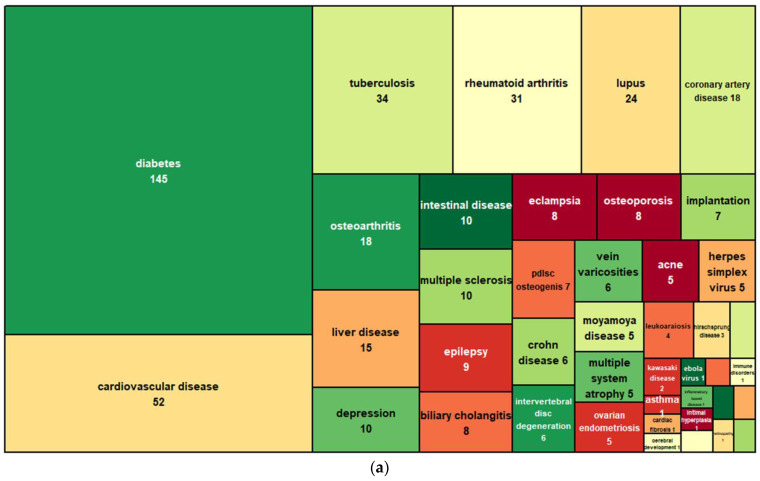

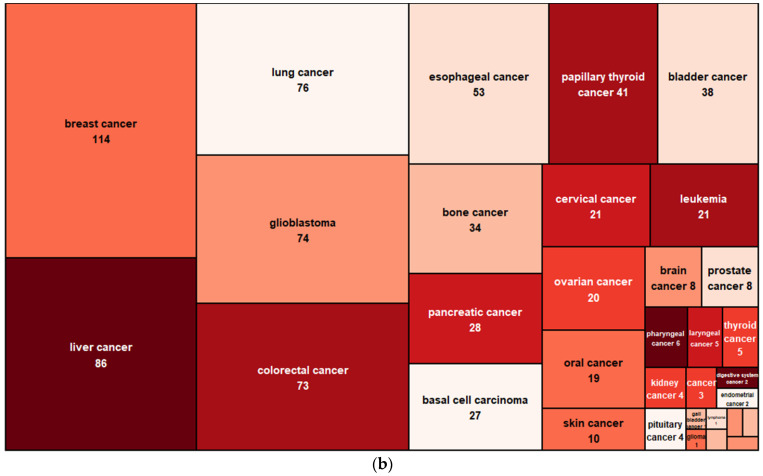
CircRNAs associated with noncancer (**a**) and cancer (**b**) diseases. Data are based on the CirCAD database [10].

CircRNAs have unusual expression profiles that are distinct from those of linear RNAs. Most circRNAs are expressed at much lower levels, usually being about 2–10% of the linear mRNAs in the cells. However, it is also common to have certain circRNAs that express at levels even higher (sometimes 10 times more) than the linear mRNAs in a cell type. For example, circRNA HIP-K3 is several times higher than linear HIP-K3 RNA [4]. Strikingly, circRNA ciRS-7 is overwhelmingly expressed in the brain, at a level of about five times that of the housekeeping gene GAPDH. Another extreme example is circRNA-SRY, which is the topmost abundant transcript in the mouse testis [9]. Compared with linear RNAs, circRNAs are more stable, as circRNAs do not have free 5′ and 3′ ends for exonuclease degradation. Whereas the half-lives of several surveyed linear RNAs are less than 20 h, their corresponding circRNA half-lives are longer than 48 h [4]. Increasingly, circular RNAs are being found to be aberrantly expressed in diseases such as cancer, as well as in cardiovascular, neurological, metabolic, or immune diseases (Figure 2). The potentially more specific disease association, stabilized presence, and compacted and facilitated release into biofluids endow circRNAs with many desired properties for new biomarker applications.

The biological functions of circRNAs are under active research. CircRNAs are mostly localized in the cytoplasm. Due to the lack of a 5′ cap for protein translation initiation, the majority of circRNAs are not usually loaded onto ribosomes and do not translate proteins [4,5]. However, some circRNAs possess internal ribosome entry sites (IRES) or RNA modifications (m6A) to translate proteins or peptides [11,12,13]. Thus, circRNAs are largely, but not entirely, noncoding RNAs. Early on, some circRNAs were observed to contain miRNA binding sites, interact with microRNAs, and functionally form complexes with an AGO-silencing complex. In particular, circRNA ciRS-7 harbors 73 miR-7 binding sites but is completely resistant to miRNA-mediated target destabilization. It acts as a “microRNA sponge” to strongly sequester miRNA gene silencing and functionally control midbrain development [3]. Another early example of circRNAs acting as a microRNA sponge is circSRY, a circRNA that is predominantly expressed in the mouse testis from the SRY gene for male sex determination. CircSRY has 16 miR-138 binding sites and functionally regulates the luciferase/SRY reporter gene expression mediated by miR-138 [14]. By analyzing the single nucleotide polymorphism (SNP) densities in circular RNAs, the miRNA binding seed regions of the circRNAs are depleted of SNPs over the linear RNA background [15]. These density sinks are right on the miRNA binding sites, suggesting the miRNA binding sites are conserved and functional. Therefore, circRNAs acting as microRNA sponges may not be merely isolated cases, particularly when the circRNAs are associated with AGO complex, and the stoichiometry of circRNA vs. mRNA binding sites can support such a function. Today, quite a number of circRNAs have been observed to function as microRNA sponges in diverse biological roles as will described later.

**Figure 3:**
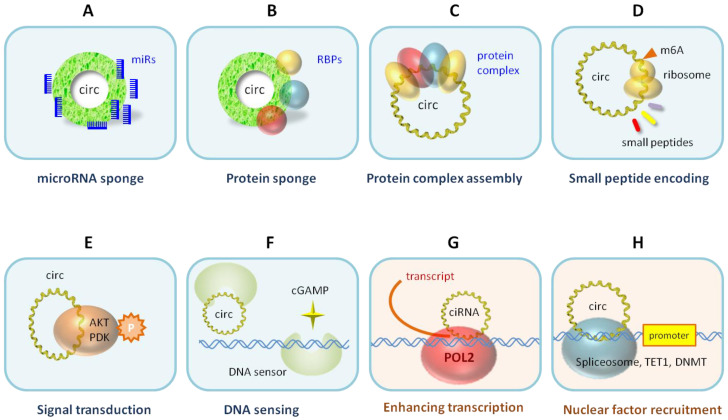
Molecular functions of circRNAs. CircRNAs are localized in the cytoplasm (**A**–**F**) or in the nucleus (**G**,**H**) to exert their molecular functions.

Beyond the microRNA sponge function (Figure 3A), surprising new circRNA molecular functions were discovered during the studies of differentially expressed circRNAs (Figure 3). Similar to miRNA sponging, circRNAs can bind proteins as RNA-binding protein (RBP) sponges (Figure 3B). There are several hundred RBPs that do a variety of biological functions, e.g., transcription, splicing, RNA processing, RNA transport, RNA localization, translation, RNA stability, and RNA modification. Over the years, circRNAs have been found to be involved in many of these RBP-related functions. CircRNAs can also be the guiding scaffold for protein complex assembly (Figure 3C). The circRNA and protein interactions have been cataloged in the circInteractome database [16,17]. Although circRNAs generally do not translate proteins due to the lack of the 5′ cap structure for translation initiation, some circRNAs can initiate protein or small peptide translation from the IRES or the internal m6A modification that recruits a translation initiation complex (Figure 3D). Some of these small peptides can be bioactive, e.g., as competing dominant negative inhibitors. Increasingly, circRNAs are found to interact with intracellular signaling molecules, e.g., AKT and PDK, to modulate signal transduction [18] (Figure 3E). Interestingly, the circRNA cia-cGAS can act as a DNA sensor switch to control the secondary messenger cyclic GAMP [19] (Figure 3F). In the nucleus, intronic circRNAs can tether RNA polymerase II to enhance the transcription [7] (Figure 3G), spliceosomes for splicing, or chromatin modifiers (e.g., TET1 and DNMTs) to epigenomically regulate gene expression (Figure 3H).

The unusual circular structure, extraordinary expression patterns, novel biological functions, disease associations, and stabilized presence in biofluids for biomarker uses have sparked immediate and intense scientific interest. CircRNA transcriptome-wide differential expression profiling in basic or clinical science is a key step in providing insight into how the circRNA expression is perturbed, predicting the biological consequences of the circRNA changes, and identifying circRNA leads for diagnostic/prognostic biomarkers. The circRNA microarrays have been a very sensitive, effective, and practical method of circRNA profiling. By directly interrogating circular junctions with the array probes, the expression of circRNAs is sensitively and accurately quantified. Numerous discoveries have been made based on the circRNA profiling studies. Some very exciting ones are highlighted here.

## 2. CircRNA Expression Profiling by Microarray

Circular RNAs are detected primarily through the circular junction sequences unique to them, by using microarrays, sequencing, qPCR, Northern blot, or fluorescence in situ hybridization (FISH). CircRNA microarrays and circRNA sequencing are the two high-throughput technologies capable of transcriptome-wide circRNA profiling. For circRNA expression profiling, the circRNA microarray is a more sensitive, accurate, convenient, and practical choice. Arraystar circRNA microarrays (Arraystar, Inc., Rockville, MD, USA) have been widely used and are taken here as the example.

The circRNA microarray contents are collected from published circRNA studies and public databases (Table 1). CircRNAs from these sources were qualified based on the adopted confidence scorings.

**Figure 4:**
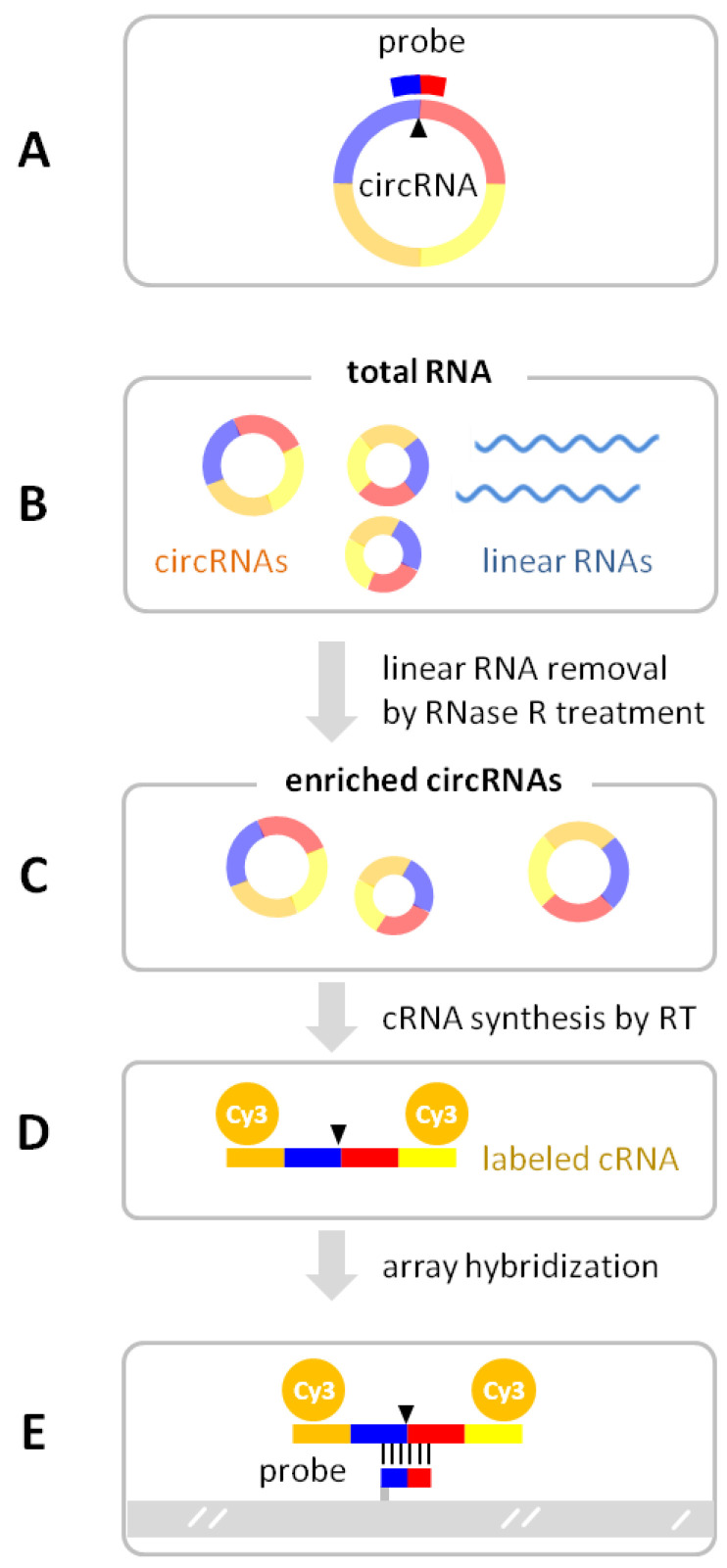
CircRNA microarray detection of circRNAs. (**A**) The array’s probe sequence spans the circular junction (arrow) of the circRNA. In the circRNA microarray workflow, total RNA (**B**) is treated with RNase R to selectively digest the linear RNAs so as to enrich the circRNAs (**C**). The circRNAs are copied by reverse transcriptase (RT) from the annealed random primers containing a T7 promoter. The cRNA is synthesized from the T7 promoter at the end of the cDNA template by in vitro T7 polymerase transcription, incorporating fluorescent Cy3-UTP (**D**). The fluorescent cRNA is hybridized to the probes printed on the array surface (**E**).

The circRNA microarrays detect circular junctions by using probe sequences that hybridize with the circular junction sequences in the circRNAs (Figure 4A). In the workflow (Figure 4B–E), ~1 µg of total RNA is used as the starting material. As there are hundreds of circRNAs on the circRNA microarrays that are smaller than 200 nt, the total RNA purification method must recover both small (< 200 nt) and large (≥ 200 nt) RNAs. Standard TRIzol Reagent method or RNA purification kits certified for both small and large RNAs should work well. The total RNA samples are measured for RNA concentrations and amounts using a Nanodrop spectrophotometer, with OD260/280 being close to 2 and OD260/230 > 1.8. For the best results, RNAs should be of good integrity, which can be checked by either denaturing RNA agarose gel electrophoresis as sharp and bright 18S and 28S rRNA bands, or by using a Bioanalyzer (RIN > 7). Degraded RNA samples can accumulate nicks or cleavages that can linearize circular RNAs, which will be destroyed during the RNase R treatment step. As a single nick or breakage in a circRNA strand may not be visible in decreased sizes, RNA sample QC should be mindful about the possibility that RNA samples appearing intact may still have varying degrees of underlying circRNA lesions or RNase contamination still active during the RNase R treatment. It was observed that cells that are stored frozen for different time periods can accumulate circRNA lesions and have altered circRNA profiles [20].

The total RNA is first treated with linear RNA specific RNase R (Epicentre Biotechnologies, Madison, WI, USA) to selectively digest and remove the linear RNAs, while leaving the circRNAs intact. The RNase R is certified by the manufacturer as being endonuclease-free. CircRNA enrichment efficiency (i.e., circ/linear ratio increase) by RNase R is typically 10-fold. The enriched circRNAs are reverse-transcribed into cDNAs from the random primers containing a T7 promoter. Thousands of copies of complementary RNAs (cRNA, or aRNA for antisense RNA) are transcribed from the T7 promoter via strong T7 polymerase in vitro, where fluorescent Cy-3 UTP is incorporated into the cRNA strands (Arraystar Super RNA Labeling Kit, Arraystar, Inc., Rockville, MD, USA) in the Eberwine linear amplification process [21,22]. Compared with exponential amplification through PCR, the linear RNA amplification retains much higher fidelity to the native RNA abundances. As RNA:DNA binding is stronger than DNA:DNA, the hybridization signals with single-stranded cRNA targets are much better than DNA targets. For circRNA microarray hybridization, the labeled cRNA is fragmented in the chemical fragmentation buffer at 60 °C for 30 min, quenched, and diluted in the hybridization buffer. An amount of 50 μL of the cRNA hybridization solution is applied to an array on the circRNA microarray slide. The slide is incubated at 65 °C for 17 h in an Agilent hybridization oven. After washing, the microarray is scanned with an array scanner (Agilent Scanner G2505C, Agilent Technologies, Inc., Santa Clara, CA, USA) and the data points are extracted from the scanned image using Agilent feature extraction software (version 11.0.1.1).

The QC processes are implemented to ensure data quality. In addition to the RNA sample quality requirements, the amplified cRNA amount must be > 1.65 µg and its dye labeling specific activity (pmol dyes per µg cRNA) > 9. The overall array signal quality is evaluated with the Agilent array QC system implemented in the scanner software and the QC metrics. Among all the QC metrics, circRNA arrays normally generate lower raw intensities compared to linear RNA microarrays as circular RNAs, as a population, are typically present at much lower abundance levels (about 2–10% of linear RNAs). For the array that passed the QC, each circRNA probe signal is flagged as P (present), A (absent), or M (marginal) (Table 2).

The microarrays are manufactured and quality controlled to excellent technical specifications: sensitivity of detecting rare transcripts as low as 1 target in 125 million unrelated sequences or 1 transcript per cell; dynamic range of 5 logs of magnitudes to detect both high- and low-expressing genes; and high technical reproducibility, with interarray correlation of technically replicated reference at R2 > 0.96.

## 3. CircRNA Microarray Analysis and Annotation

CircRNA microarray raw intensity data are normalized as log 2 transformed “normalized intensities” using a quantile normalization method implemented in R-limma package [23,24]. The normalized intensities are used to represent the relative circRNA abundance levels and for differential expression analysis. The statistically significant, differentially expressed circRNAs are filtered using FC (abs) and *p*-value cutoffs (Table 3). False-discovery rates (FDRs) are computed using the Benjamini–Hochberg procedure to account for multiple tests on all the circRNA comparisons and to estimate the statistical false-positive rates among the differentially expressed genes. Volcano plots, scatter plots, and hierarchical clustering heatmaps are generated to visually aid in the identification of the differentially expressed circRNAs of interest. Typically, circRNAs with large FCs and small *p*-values on the volcano plot have the best chances of being confirmed as differentially expressed by qPCR. CircRNAs at higher abundance levels and with bigger differences in the scatter plot are more likely to produce biological effects (e.g., in miRNA sponge stoichiometry).

Some data fields in the table (not shown here) include:

- Database source
- Chromosomal coordinates
- Host gene
- Best transcript
- CircRNA type (Figure 1)
- CircRNA length
- Probe sequence
- Top five miRNA binding sites

To gain biological insights, the functions of the differentially expressed circRNAs are bioinformatically predicted and annotated. As exonic, sense-overlapping, and antisense circRNAs (Figure 1) share parts of the sequences of their mRNA counterparts in either in sense or antisense directions, they may exert regulatory effects on these mRNAs, e.g., as competing endogenous RNAs [25,26,27] or by direct antisense base pairing between the circRNA and mRNA. The biological outcomes of the differentially expressed circRNAs can be predicted via their targeted mRNA functions through gene ontology (GO) and pathway enrichment analyses, using topGo R-package [28], DAVID [29,30], or a number of other software tools.

**Figure 5:**
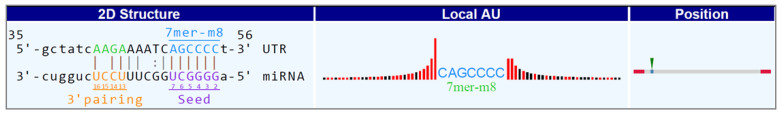
An example of circular RNA and its binding miRNA. The MRE type (7mer-m8), seed region, 3′ pairing, local AU, and position in the circRNA are displayed.

Potential miRNA sponge functions are analyzed for miRNA binding sites on the circRNAs by using both miRanda [31] and TargetScan [32,33]. Top-scoring microRNA response elements (MRE) are graphically displayed for their MRE types, seed regions, 3′ pairing, local AU, and binding site positions in the corresponding circRNAs (Figure 5). The physical and functional circRNA–miRNA interaction can be experimentally tested by, for example, using circRNA-qPCR on the biotinylated miRNA pull down or anti-AGO immunoprecipitation.

## 4. circRNA Biomarkers in Biofluids and Exosomes

Being circularized to resist exonuclease degradation, being more stable in biofluids, and having higher cell/tissue/disease specificity, circRNAs have been explored as a new class of biomarkers, with the motivation of finding biomarkers that are potentially superior to the existing ones in other molecular classes [34,35].

One common challenge with biofluid samples (e.g., serum, plasma, or exosomes) for circRNA expression profiling is that the RNA amounts are often highly variable and very low. It is important to prepare enough starting materials so as to obtain sufficient total RNAs for the circRNA microarray and follow-up studies (Table 4).

RNAs in biofluids or exosomes are always heavily fragmented and degraded, which also subject the circRNAs in the samples to increased probability of RNA strand breakage, linearization, and decay. The approach here is to obtain the topmost differentially expressed circRNAs that can still survive in the condition of the biofluid for microarray signals, while being realistic about the overall profiling data for the rest of the circRNAs. The identified differentially expressed circRNAs should be confirmed by circRNA-qPCR, which has the higher sensitivity, better quantification accuracy, and no signal loss due to circRNA linearization and RNase R digestion (not used in qPCR). Many biofluid circRNAs for biomarkers have been successfully identified (Table 5).

## 5. Confirmation of Differentially Expressed circRNAs

**Figure 6:**
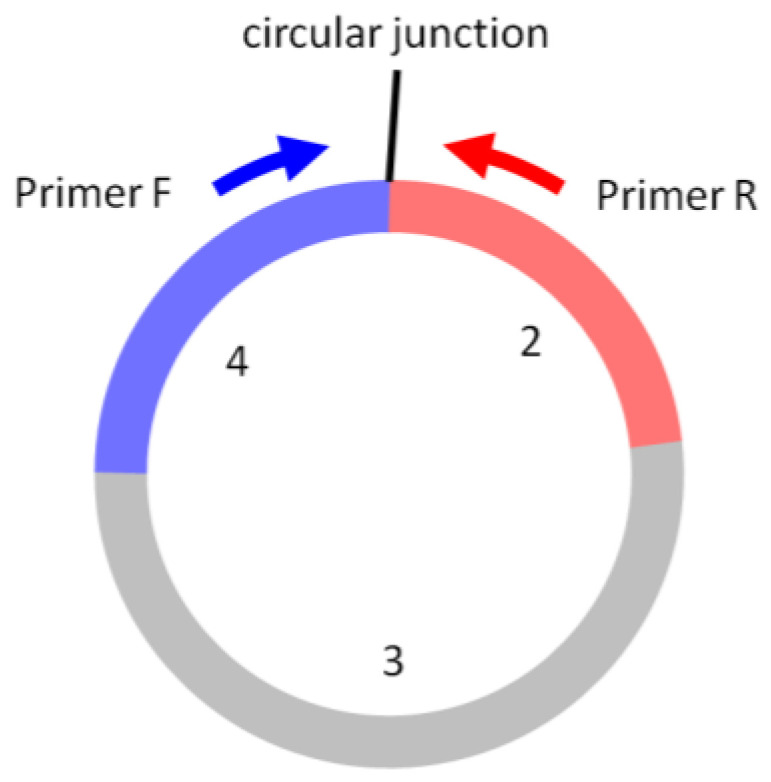
In circRNA-qPCR, Primer F and Primer R are situated on the two sides of the circular junction, producing the amplicon that spans the circular junction. The exon numbers are for illustration only.

CircRNA-qPCR is commonly used as an independent and quantitatively accurate method to confirm the differentially expressed circRNAs identified in the circRNA expression profiling [2,4]. CircRNA-qPCR by SYBR Green chemistry is technically very similar to regular linear mRNA qPCR, with the following important considerations:

- The forward and reverse circRNA-qPCR primers should be situated separately on the two sides of the circular junction, such that the amplicon spans the circular junction (Figure 6). The primer sequence and amplicon design can follow the same guidelines for linear RNA qPCR using a software tool (e.g., Primer3Plus).
- Circular RNAs do not have a poly(A) tail. Random RT primers, instead of oligo(dT), are used in the first-strand cDNA synthesis.
- An amount of 0.5–2 µg of total RNA is used as the starting material. RNase R treatment for circRNA enrichment is not necessary and not performed. The synthesized cDNA can be used for both circRNA and linear mRNA qPCR.
- Linear housekeeping genes, such as GAPDH, ACTB, B2M, or 18S rRNA, can continue to be used normally as the qPCR normalization reference. For biofluid samples, the average of more than one housekeeping gene or the input biofluid volumes can also be used for the normalization reference. Currently, no known circRNAs have been qualified as “housekeeping” circRNAs that are stably expressed at high levels across all tissues and cell types.
- Post-qPCR melt curve analysis should be used to ensure the specific amplification and the absence of primer dimers.
- *Optional*: The qPCR product can be run on a gel to verify if the DNA is a specific single band and if its size is in agreement with the predicted amplicon size.
- *Optional*: The qPCR product can be cleaned up and Sanger-sequenced for circular junction sequence confirmation.
- *Optional*: Circular:linear ratios can be compared in parallel qPCRs with and without RNase R treatment, using primers that are specific for circular RNA sites only and for linear RNA sites only. An increase in the circular:linear ratio by RNase R treatment is a strong indication of the RNA being circRNA.
- *Caveats*: Template switching during the reverse-transcription step of cDNA synthesis can occur, which can produce spuriously joined exons as “circRNAs” and false-positive signals [36].

A circRNA is considered to be present when Ct < 35, or absent when Ct ≥ 35. The confirmation rate depends on the FC, *p*-value, and abundance of the selected circRNA. Using the same RNA sample for the circRNA profiling and qPCR increases the concordance.

## 6. Highlights of circRNAs Identified on circRNA Microarrays

Differentially expressed circRNAs identified on the circRNA microarrays have been further studied for their biological functions, clinical indications, molecular mechanisms, and biomarker applications. About 500 papers have been published using the circRNA microarrays so far.

Many identified circRNAs have been analyzed for their miRNA sponge functions (Table 6), some of which were experimentally supported.

Beyond the miRNA sponge functions, the circRNAs identified in the circRNA microarray profiling studies have been discovered performing surprising functions, some of which are highlighted here:

*Protein binding* (Figure 3B,C). CircRNA FNDC-3b was among the top differentially expressed circRNAs in myocardial infarction [37]. It binds and sequestrates apoptosis tumor suppressor protein FUS-1, resulting in increased VEGF levels and enhanced endothelial function in myocardial infarction. Adeno-associated virus (AAV9) gene delivery of circFNDC-3b therapeutically improves the postmyocardial infarction recovery.

*DNA sensing* (Figure 3F). CircRNA ciaRNA was identified as being differentially expressed between hematopoietic stem cells under homeostasis and cell cycling [19]. ciaRNA binds cyclic GMP-AMP synthase (cGAS) to block the cGAS interaction with DNA fragments. Without ciaRNA, cGAS-DNA sensing is turned on to generate secondary messenger cyclic GAMP, which activates the production of interferon. Thus, ciaRNA is a potent suppressor of cGAS-mediated DNA sensing for cell homeostasis and autoimmune response.

*Signal transduction* (Figure 3E). CircRNA circ-AMOTL1 was identified as being differentially expressed between neonatal and mature cardiac tissue [18]. It interacts with serine/threonine protein kinase AKT1 and pyruvate dehydrogenase kinase PDK1. The binding leads to phosphorylation of the kinases, nuclear transport, and enzymatic activation, which reduces apoptosis and repairs cardiac damage.

*Nuclear transcription* (Figure 3G). CircRNA circACTN4 was identified as a top differentially expressed circRNA in cholangiocarcinoma [38]. In the nucleus, circACTN4 recruits YBX1 protein to activate the FZD7 gene promoter for the frizzled WNT receptor. FZD7 in the Wnt/β-catenin pathway then activates YAP1 in the Hippo pathway to cause cancer growth and metastasis. CircACTN4 also binds another nuclear protein, FUBP1, to activate the MYC oncogene and promote breast cancer [39]. CircACTN4 is also a biomarker for poor cancer prognosis and faster postsurgery cancer recurrence [38]. In another example, circRNA circIPO11 interacts with the DNA topoisomerase TOP1 protein to target and activate the transcription factor Gli gene promoter, consequently driving the Hedgehog pathway and hepatocellular carcinoma [40].

*Epigenomic regulation* (Figure 3H). The circRNA for Scm polycomb group protein homolog 1 was identified as being differentially expressed in ischemic strokes [41]. The circRNA binds to the methyl-CpG binding protein (MeCP2), which relieves the CpG island methylation and activates the target genes. The recombinant circRNA treatment improved the functional recovery after a stroke in mice and monkeys.

In addition to functional studies, many differentially expressed circRNAs identified by the circRNA microarrays have been actively studied for biomarker uses. Published biomarker studies that used plasma, serum, or exosomal samples are listed (Table 5).

## 7. CircRNA Microarray Advantages Compared with Sequencing

Compared with other methods that can quantify limited numbers of circRNAs (e.g., qPCR, Northern blot, NanoString, FISH), circRNA microarrays and circRNA-seq have the capacity and throughput to quantitatively profile circRNAs transcriptome-wide. CircRNA-seq has been instrumental in discovering and identifying circRNAs de novo. CircRNA-seq requires circRNA enrichment from the total RNA through rRNA removal, RNase R treatment, or polyadenylation counter-selection [42,43]. Even with the enrichment, deeper sequencing coverage is needed to detect the circular junctions that are typically in low abundance. CircRNA-seq relies on mapping the circular junction sequences by using specially developed tools, such as CIRCexplorer2 [44], circRNA_finder [45], CIRI2 [46], find_circ [3], and Mapsplice [47], using either de novo analysis or a preconstructed database of all the possible back splices built on gene exon models [1,2,48]. The circular splice junction mapping methods are complicated and are still less reliable [2]. Currently, no single individual pipeline can accurately annotate all of the circRNAs. Instead, integrating multiple pipelines and RNase R experiments has been studied to gain better results [49]. Additionally, RT template-switching and ligation artifacts during the circRNA-seq library construction can produce artifactually joined cDNA sequences that have been falsely identified as “circRNAs” [36]. At the end, the identified circRNAs can be classified as high-, medium-, and low-confidence/stringency circRNAs based on their repeatable occurrences in one or more samples [4,36].

Once the circRNAs are identified and cataloged, the most common objective of circRNA profiling is the quantitative analysis of their differential expression, for example, between conditions of experiment vs. control, disease vs. healthy, or after- vs. before-treatment. Whereas using circRNA-seq for quantitative profiling has many limitations, circRNA microarray has many technical and practical advantages.

**Figure 7:**
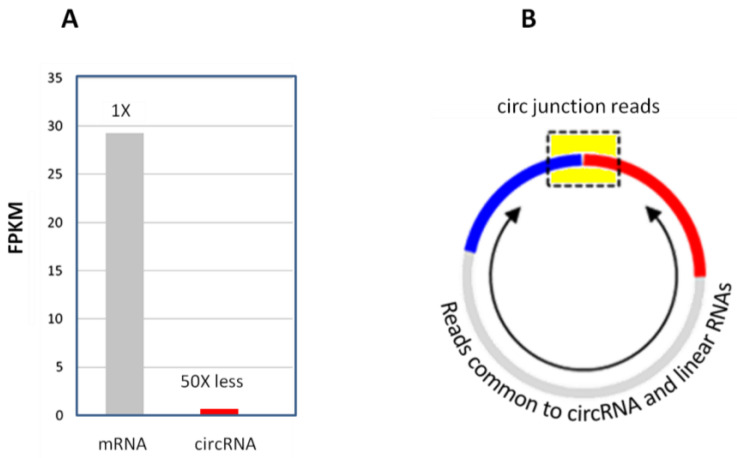
(**A**) CircRNA abundance relative to mRNA in total RNA. (**B**) Circular junction reads (in the yellow box region) only represent a small portion of the entire circRNA. The reads from other parts of the circRNA body (arrowed region) are indistinguishable from that of the linear RNA counterparts.

One physical challenge for circRNA-seq is the poor sequencing read coverage for circular junctions. CircRNAs, as a population, are generally expressed at much lower abundance levels, being about 1–3% of the linear mRNAs (Figure 7A) [2]. Unlike linear mRNA-seq, where sequencing reads for any part of the mRNA transcript are counted toward that mRNA, circRNA-seq only counts the circular junction reads (Figure 7B). Sequencing reads in other parts of the circRNA body cannot be distinguished from its linear RNA isoforms and are disregarded. That is, the circular junction reads represent only a small proportion of the circRNA sequencing reads. These factors made circular junction read counts very low in most circRNA-seq studies, even at very deep sequencing coverage. For example, prior circRNA studies often observed just a few low counts of circRNAs (Table 7).

**Figure 8:**
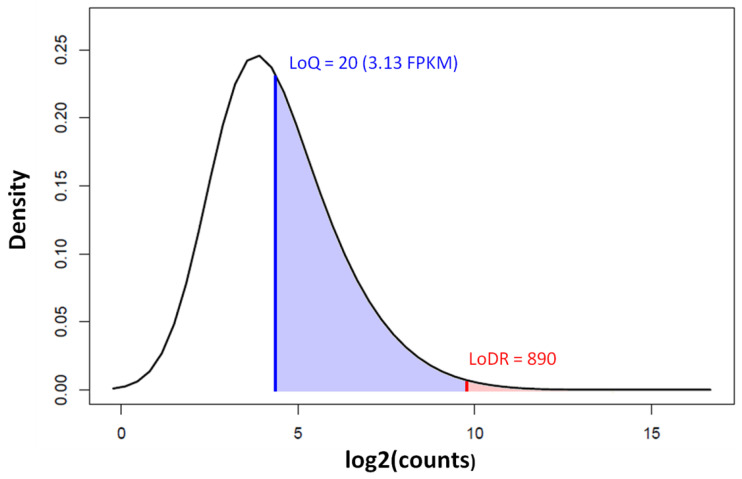
Density distribution of circRNA counts by circRNA-seq in colorectal cancer and normal control samples (GEO Accession: GSE205241). The limit of quantification (LoQ) is indicated by the blue vertical line and the limit of differential ratio (LoDR, down to a 2-fold change) by the red vertical line. Less than 0.3% of the circRNAs are above the LoDR (shaded red). The circRNA-seq was run on Illumina NovaSeq 6000 at a 150 bp read length and a coverage of 40 million paired end fragments.

The fundamental difficulty of the low read counts in circRNA-seq is the inability to conduct a quantitative analysis. For the mere detection of a circRNA’s presence (limit of detection, LoD), a few repeated observations of the circRNA junction are sufficient. However, for the quantification of transcript abundance, the minimum RNA abundance required for a sufficiently accurate measurement (limit of quantification, LoQ) is 3.13 FPKM [50], which is about 20 circular junction counts in a sequencing run, at a read length of 150 and a coverage of 40 million reads. For differential expression analysis between comparison groups, the minimum read count required for detecting the differential expression down to a 2-fold change (limit of differential ratio, LoDR) is about 890 [50]. Very few circRNAs can reach the abundance levels above the LoDR that are required to be detected as being differentially expressed (Figure 8).

Along with the less-established and more complicated analysis pipeline, the quantification inaccuracy and high false-positive issues are still not fully resolved [36]. In fact, the same RNA samples profiled by different circRNA-seq library preparation methods and analysis algorithms show very low concordance and very large discrepancies [51]. On a practical level, circRNA-seq pipelines are harder to set up and are less accessible to biologists and clinicians whose main interests are in biology, not circRNA computation.

**Figure 9:**
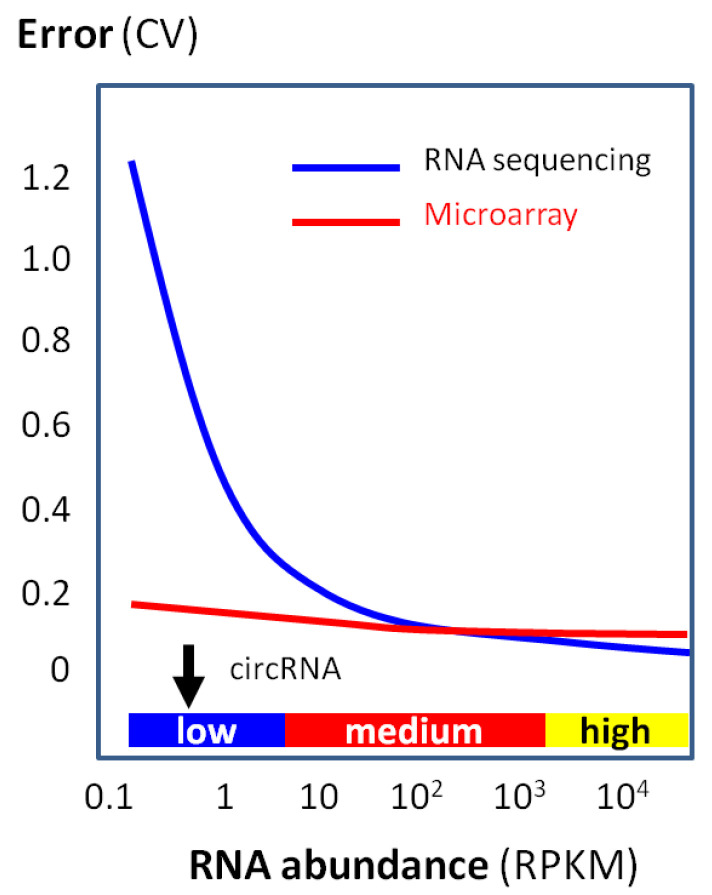
Quantification accuracy (i.e., low error) with RNA abundance by using a microarray or RNA sequencing. Data are based on a large-scale clinical study [52]. While RNA sequencing rapidly loses accuracy (i.e., high error) when the RNA abundance is low, microarray accuracy is relatively unaffected even with a low RNA abundance. Typical circRNA abundance is indicated by the black arrow.

On the other hand, circRNA microarray profiling is relatively unaffected by the low RNA levels and maintains the accuracy very well for RNAs, even in low abundance (Figure 9).

Using public datasets, the differentially expressed circRNAs in human colorectal cancer and normal tissues (*n* = 3 per group) are compared using the circRNA microarray and circRNA-seq (Table 8). The differentially expressed circRNAs are filtered by the same stringency cutoff at |FC| ≥ 2.0, *p* ≤ 0.05, and the abundance ≥ LoDR for the platforms. The microarray found >35× more differentially expressed circRNAs.

Overall, circRNA arrays are much more efficient, sensitive, and accurate in circRNA expression profiling (Table 9). For lab biologists and clinical researchers, circRNA microarray profiling provided by a specialized service is a more practical way than tweaking RNA-seq for circRNA-seq, which has generated hundreds of scientific publications in circRNA research.

## Figures and Tables

**Table 1:** CircRNA collections on the circRNA microarrays.

	Human	Mouse	Rat
**CircRNA microarray**	Human CircRNA microarray V2	Mouse CircRNA microarray V2	Rat CircRNA microarray V1
**GEO platform ID**	GPL21825	GPL21826	GPL21828
**CircRNA probes**	13,617	14,236	14,145
**Probe length**	60 nt		
**Probe sequence**	Targeting the circular junctions		
**Labeling method**	CircRNA enrichment by RNase R treatment; Random priming for reverse transcription; Linear cRNA amplification by T7 polymerase transcription in vitro; Fluorescent labeling by C3-UTP incorporation.		
**Array Format**	8 × 15K	8 × 15K	8 × 15K
**circRNA sources**			
Salzman’s circRNAs [2]	8529		
Memczak’s circRNAs [3]	1601	1750	
Zhang’s circRNAs [7]	93		
Zhang’s circRNAs [6]	4980		
Jeck’s circRNAs [4]	3769		
Guo’s circRNAs [5]	5536	570	
You’s circRNAs [8]		13,300	12,298
Mouse orthologs			1668
Human orthologs			179

**Table 2:** CircRNA probe signal QC flags.

QC flag	P (Present)	A (Absent)	M (Marginal)
Feature is not positive and significant		X	
Feature is not uniform		X	
Feature is not above background		X	
Feature is saturated		X	
Feature is population outlier		X	
Background is not uniform			X
Background is population outlier			X

**Table 3:** Snapshot of differentially expressed circRNA table.

circRNA	ProbeID	*p*-Value	FDR	FC (Abs)	Regulation
hsa_circRNA_104264	ASCRP3004838	0.00002735	0.001493	6.955	up
hsa_circRNA_001593	ASCRP3007121	0.00009951	0.005795	3.627	down
hsa_circRNA_007223	ASCRP3010883	0.00006019	0.003864	1.774	down

**Table 4:** Biofluid volumes for circRNA microarray.

Biofluid	Liquid Volume	Typical Total RNA Yield *	Starting Vol. for Exosome Isolation
Whole blood	0.5 mL or 2 million blood cells	tens of micrograms	
Plasma	1–2 mL	300–1500 ng/mL	2 mL
Serum	1–2 mL	300–1500 ng/mL	2 mL
Saliva	200 μL	0.9–7 µg	
Amniotic fluid	1–2 mL	4 µg/mL	
Cell culture medium	50 mL	20 ng/mL	50 mL
Urine			50 mL

* The yields are highly variable.

**Table 5:** Published biofluid biomarker studies based on circRNA microarray profiling.

Sample Type	Title	Journal	Pubmed
plasma	Circular RNA in blood corpuscles combined with plasma protein factor for early prediction of pre-eclampsia.	BJOG (2016)	26846540
plasma	Microarray profiling and functional analysis reveal the regulatory role of differentially expressed plasma circular RNAs in Hashimoto’s thyroiditis.	Immunol Res (2022)	35064448
plasma	Circular RNA expression profiles in the plasma of patients with infantile hemangioma determined using microarray analysis.	Exp Ther Med (2021)	33968165
plasma	Plasma circular RNA panel acts as a novel diagnostic biomarker for colorectal cancer detection.	Am J Transl Res (2020)	33312376
plasma	Identification of plasma hsa_circ_0008673 expression as a potential biomarker and tumor regulator of breast cancer.	J Clin Lab Anal (2020)	32808350
plasma	Plasma hsa_circ_0027089 is a diagnostic biomarker for hepatitis B virus-related hepatocellular carcinoma.	Carcinogenesis (2020)	31535687
plasma	Selective downregulation of distinct circRNAs in the tissues and plasma of patients with primary hepatic carcinoma.	Oncol Lett (2019)	31612035
plasma	Circulating plasma circular RNAs as novel diagnostic biomarkers for congenital heart disease in children.	J Clin Lab Anal (2019)	31429492
plasma	Using plasma circRNA_002453 as a novel biomarker in the diagnosis of lupus nephritis.	Mol Immunol (2018)	30172209
plasma	Dysregulated circRNAs in plasma from active tuberculosis patients.	J Cell Mol Med (2018)	29961269
serum	Plasma circular RNA profiling of patients with gastric cancer and their droplet digital RT-PCR detection.	J Mol Med (2018)	29098316
serum	Effects of renal denervation on the expression profile of circular RNA in the serum of patients with resistant hypertension.	Hellenic J Cardiol (2022)	34147676
serum	Correlation between serum circRNA and thyroid micropapillary carcinoma with cervical lymph node metastasis.	Medicine (2020)	33217846
exosomes	Exosomal circCARM1 from spheroids reprograms cell metabolism by regulating PFKFB2 in breast cancer.	Oncogene (2022)	35027669
exosomes	Serum exosomal hsa_circ_0069313 has a potential to diagnose more aggressive non-small cell lung cancer.	Clin Biochem (2022)	35077682
exosomes	Exosomal hsa_circRNA_104484 and hsa_circRNA_104670 may serve as potential novel biomarkers and therapeutic targets for sepsis.	Sci Rep (2021)	34238972
exosomes	Hypoxic exosomal HIF-1α-stabilizing circZNF91 promotes chemoresistance of normoxic pancreatic cancer cells via enhancing glycolysis.	Oncogene (2021)	34294845
exosomes	Tumor-derived exosomal circRNA051239 promotes proliferation and migration of epithelial ovarian cancer.	Am J Transl Res (2021)	33841644
exosomes	Identification of low-dose radiation-induced exosomal circ-METRN and miR-4709-3p/GRB14/PDGFRα pathway as a key regulatory mechanism in Glioblastoma progression and radioresistance: Functional validation and clinical theranostic significance.	Int J Biol Sci (2021)	33867829
exosomes	Exosomal circEhmt1 Released from Hypoxia-Pretreated Pericytes Regulates High Glucose-Induced Microvascular Dysfunction via the NFIA/NLRP3 Pathway.	Oxid Med Cell Longev (2021)	33815662
exosomes	Exosomal circular RNA_400068 promotes the development of renal cell carcinoma via the miR-210-5p/SOCS1 axis.	Mol Med Rep (2020)	33173957
exosomes	Microarray profiling and functional analysis of differentially expressed plasma exosomal circular RNAs in Graves’ disease.	Biol Res (2020)	32727578
exosomes	CircRNA-0077930 from hyperglycaemia-stimulated vascular endothelial cell exosomes regulates senescence in vascular smooth muscle cells.	Cell Biochem Funct (2020)	32307741
exosomes	Exosome circ_0044516 promotes prostate cancer cell proliferation and metastasis as a potential biomarker.	J Cell Biochem (2020)	31625175
exosomes	Abnormal expression of circRNA_089763 in the plasma exosomes of patients with post-operative cognitive dysfunction after coronary artery bypass grafting.	Mol Med Rep (2019)	31524256
exosomes	Tumor-released exosomal circular RNA PDE8A promotes invasive growth via the miR-338/MACC1/MET pathway in pancreatic cancer.	Cancer Lett (2018)	29709702
exosomes	Circular RNA IARS (circ-IARS) secreted by pancreatic cancer cells and located within exosomes regulates endothelial monolayer permeability to promote tumor metastasis.	J Exp Clin Cancer Res (2018)	30064461

**Table 6:** Differentially expressed circRNAs from circRNA microarray profiling, analyzed for miRNA sponge functions (published in years 2020–2022).

circRNA	microRNA	Effect mRNA	Indications	Pubmed
circACTN4	miR-424-5p	YAP1	Cancer	34509526
Hsa_circ_0003258	miR-653-5p	YAP1	Cancer	34986849
circDLG1	miR-141-3p	CXCL12	Cancer	34911533
circRNF220	miR-30a	MYSM1, IER2	Leukemia	34702297
circMEMO1	miR-106b-5p	TET	Cancer	33985545
circHIPK3	miR-29b	Rac1, Cdc42, cyclin B1	Repair of intestinal epithelium	34116030
circEXOC7	miR-149-3p	CSF1	Renal cell carcinoma	33783995
circNFXL1_009	miR-29b	KCNB1	Pulmonary hypertension	33614247
circ-HMGA2	miR-1236-3p	ZEB1	Lung adenocarcinoma	33762580
circ-METRN	miR-4709-3p	GRB14	Glioblastoma	33867829
circANAPC2	miR-874-3p	SMAD3	Endochondral ossification	33713570
circ-ANXA7	miR-331	LAD1	Lung adenocarcinoma	33536022
circRIMS2	miR-186	BDNF	Neuronal apoptosis	33413076
circ_0077755	miR-182		Breast cancer	33514777
circ_0071036	miR-489		Pancreatic cancer	33507122
circMTO1	miR-630	KLF6	Osteosarcoma	33506896
circPTCH1	miR-485-5p	MMP14	Renal cell carcinoma	32929380
circPRRC2A	miR-514a, 6776	TRPM3	Renal cell carcinoma	32292503
circCTNNA1	miR-149-5p	FOXM1	Colorectal cancer	32699205
circNRIP1	miR-629-3p	PTP4A1/ERK1	Cervical cancer	32457332
circErbB4	miR-29a-5p		VSMC migration	32514012
circ5615	miR-149-5p	TNKS	Colorectal cancer	32393760

**Table 7:** Low raw counts of a randomly picked circRNA hsa_circ_0054254 in various tissues and cells in the circRNA studies.

Tissues or Cells	circRNA Raw Counts	circRNA Study
frontal cortex	3	[50]
parietal lobe	7	
diencephalon	2	
K562	3	[2]
Gm12878	13	
Sknshra	1	
H1hesc	3	
Helas3	2	
Huvec	0	
A549	3	
Ag04450	1	

**Table 8:** Differentially expressed circRNAs by circRNA microarray and circRNA-seq.

	circRNA Microarray	circRNA-Seq
**Technology platform**	Arraystar Human CircRNA Microarray V2	Illumina NovaSeq 6000 (40 mil paired-end read coverage, 150-base read length)
**GEO Accession**	GSE159669	GSE205241
**Differential analysis method**	limma R-package (Version 3.54.1) [21]	edgeR R-package (Version 3.40.2) [53,54,55]
**Stringency cutoffs**	|FC| ≥ 2 *p* ≤ 0.05 Intensity ≥ 7.2 *	|FC| ≥ 2 *p* ≤ 0.05 Raw counts ≥ 890 (LoDR) **
**Differentially expressed circRNAs**	**150**	**4**

* Lower boundary of the linear range for microarray intensity signals based on the microarray manufacturer’s technical QC criteria. ** LoDR as established in [50].

**Table 9:** Comparison of the circRNA microarray and circRNA-seq for circRNA expression profiling.

circRNA Microarray *	circRNA-Seq
High sensitivity for finding differentially expressed circRNAs.	The numbers of circRNAs above the LoDR are small for quantitative detection of differentially expressed circRNAs.Deep sequencing coverage to get higher circRNA counts incurs high costs.Artifacts and false positives in circRNA-seq library prep.
Unambiguous, accurate, and reliable circular junction-specific array probes.	Less reliable/accurate/established computational methods for circular RNAs.
Dedicated circRNA annotation and analysis to gain insights into the circRNA biology via the specialized circRNA microarray service.	Publicly available circRNA annotations are basic.
Good for differentially expressed circRNA analysis for humans, mice, and rat. Not available for species without established cataloged circRNAs.	Good for de novo discovery of circRNAs (e.g., in species other than humans, mice and rats).

* Arraystar circRNA microarrays as the example.

## References

[1] Salzman J., Gawad C., Wang P.L., Lacayo N., Brown P.O. (2012). Circular RNAs are the predominant transcript isoform from hundreds of human genes in diverse cell types. PLOS ONE.

[2] Salzman J., Chen R.E., Olsen M.N., Wang P.L., Brown P.O. (2013). Cell-type specific features of circular RNA expression. PLoS Genet..

[3] Memczak S., Jens M., Elefsinioti A., Torti F., Krueger J., Rybak A., Maier L., Mackowiak S.D., Gregersen L.H., Munschauer M. (2013). Circular RNAs are a large class of animal RNAs with regulatory potency. Nature.

[4] Jeck W.R., Sorrentino J.A., Wang K., Slevin M.K., Burd C.E., Liu J., Marzluff W.F., Sharpless N.E. (2013). Circular RNAs are abundant, conserved, and associated with ALU repeats. RNA.

[5] Guo J.U., Agarwal V., Guo H., Bartel D.P. (2014). Expanded identification and characterization of mammalian circular RNAs. Genome Biol..

[6] Zhang X.O., Wang H.B., Zhang Y., Lu X., Chen L.L., Yang L. (2014). Complementary sequence-mediated exon circularization. Cell.

[7] Zhang Y., Zhang X.O., Chen T., Xiang J.F., Yin Q.F., Xing Y.H., Zhu S., Yang L., Chen L.L. (2013). Circular intronic long noncoding RNAs. Mol. Cell.

[8] You X., Vlatkovic I., Babic A., Will T., Epstein I., Tushev G., Akbalik G., Wang M., Glock C., Quedenau C. (2015). Neural circular RNAs are derived from synaptic genes and regulated by development and plasticity. Nat. Neurosci..

[9] Capel B., Swain A., Nicolis S., Hacker A., Walter M., Koopman P., Goodfellow P., Lovell-Badge R. (1993). Circular transcripts of the testis-determining gene Sry in adult mouse testis. Cell.

[10] Rophina M., Sharma D., Poojary M., Scaria V. (2020). Circad: A comprehensive manually curated resource of circular RNA associated with diseases. Database.

[11] Yang Y., Fan X., Mao M., Song X., Wu P., Zhang Y., Jin Y., Yang Y., Chen L.L., Wang Y. (2017). Extensive translation of circular RNAs driven by N(6)-methyladenosine. Cell Res..

[12] Legnini I., Di Timoteo G., Rossi F., Morlando M., Briganti F., Sthandier O., Fatica A., Santini T., Andronache A., Wade M. (2017). Circ-ZNF609 Is a circular RNA that can be translated and functions in myogenesis. Mol. Cell.

[13] Miao Q., Ni B., Tang J. (2021). Coding potential of circRNAs: New discoveries and challenges. PeerJ.

[14] Hansen T.B., Jensen T.I., Clausen B.H., Bramsen J.B., Finsen B., Damgaard C.K., Kjems J. (2013). Natural RNA circles function as efficient microRNA sponges. Nature.

[15] Thomas L.F., Saetrom P. (2014). Circular RNAs are depleted of polymorphisms at microRNA binding sites. Bioinformatics.

[16] Dudekula D.B., Panda A.C., Grammatikakis I., De S., Abdelmohsen K., Gorospe M. (2016). CircInteractome: A web tool for exploring circular RNAs and their interacting proteins and microRNAs. RNA Biol..

[17] Panda A.C., Dudekula D.B., Abdelmohsen K., Gorospe M. (2018). Analysis of circular RNAs using the web tool circinteractome. Methods Mol. Biol..

[18] Zeng Y., Du W.W., Wu Y., Yang Z., Awan F.M., Li X., Yang W., Zhang C., Yang Q., Yee A. (2017). A circular RNA binds to and activates akt phosphorylation and nuclear localization reducing apoptosis and enhancing cardiac repair. Theranostics.

[19] Xia P., Wang S., Ye B., Du Y., Li C., Xiong Z., Qu Y., Fan Z. (2018). A circular RNA protects dormant hematopoietic stem cells from DNA Sensor cGAS-mediated exhaustion. Immunity.

[20] Zhang Y., Huang G., Yuan Z., Zhang Y., Chen X., Huang J., Li N., Liu Z., Zhong W., Huang H. (2022). Profiling and bioinformatics analysis revealing differential circular rna expression about storage lesion regulatory in stored red blood cells. Transfus. Med. Hemother..

[21] Van Gelder R.N., von Zastrow M.E., Yool A., Dement W.C., Barchas J.D., Eberwine J.H. (1990). Amplified RNA synthesized from limited quantities of heterogeneous cDNA. Proc. Natl. Acad. Sci. USA.

[22] Li J., Eberwine J. (2018). The successes and future prospects of the linear antisense RNA amplification methodology. Nat. Protoc..

[23] Bolstad B.M., Irizarry R.A., Astrand M., Speed T.P. (2003). A comparison of normalization methods for high density oligonucleotide array data based on variance and bias. Bioinformatics.

[24] Ritchie M.E., Phipson B., Wu D., Hu Y., Law C.W., Shi W., Smyth G.K. (2015). limma powers differential expression analyses for RNA-sequencing and microarray studies. Nucleic. Acids. Res..

[25] Salmena L., Poliseno L., Tay Y., Kats L., Pandolfi P.P. (2011). A ceRNA hypothesis: The Rosetta Stone of a hidden RNA language?. Cell.

[26] Tay Y., Kats L., Salmena L., Weiss D., Tan S.M., Ala U., Karreth F., Poliseno L., Provero P., Di Cunto F. (2011). Coding-independent regulation of the tumor suppressor PTEN by competing endogenous mRNAs. Cell.

[27] Tay Y., Rinn J., Pandolfi P.P. (2014). The multilayered complexity of ceRNA crosstalk and competition. Nature.

[28] Alexa A., Rahnenfuhrer J., Lengauer T. (2006). Improved scoring of functional groups from gene expression data by decorrelating GO graph structure. Bioinformatics.

[29] Huang W., Sherman B.T., Lempicki R.A. (2009). Systematic and integrative analysis of large gene lists using DAVID bioinformatics resources. Nat. Protoc..

[30] Sherman B.T., Hao M., Qiu J., Jiao X., Baseler M.W., Lane H.C., Imamichi T., Chang W. (2022). DAVID: A web server for functional enrichment analysis and functional annotation of gene lists (2021 update). Nucleic. Acids Res..

[31] Enright A.J., John B., Gaul U., Tuschl T., Sander C., Marks D.S. (2003). MicroRNA targets in Drosophila. Genome Biol..

[32] Agarwal V., Bell G.W., Nam J.W., Bartel D.P. (2015). Predicting effective microRNA target sites in mammalian mRNAs. Elife.

[33] McGeary S.E., Lin K.S., Shi C.Y., Pham T.M., Bisaria N., Kelley G.M., Bartel D.P. (2019). The biochemical basis of microRNA targeting efficacy. Science.

[34] Wen G., Zhou T., Gu W. (2021). The potential of using blood circular RNA as liquid biopsy biomarker for human diseases. Protein Cell.

[35] Li Y., Zeng X., He J., Gui Y., Zhao S., Chen H., Sun Q., Jia N., Yuan H. (2018). Circular RNA as a biomarker for cancer: A systematic meta-analysis. Oncol. Lett..

[36] Szabo L., Salzman J. (2016). Detecting circular RNAs: Bioinformatic and experimental challenges. Nat. Rev. Genet..

[37] Garikipati V.N.S., Verma S.K., Cheng Z., Liang D., Truongcao M.M., Cimini M., Yue Y., Huang G., Wang C., Benedict C. (2019). Circular RNA CircFndc3b modulates cardiac repair after myocardial infarction via FUS/VEGF-A axis. Nat. Commun..

[38] Chen Q., Wang H., Li Z., Li F., Liang L., Zou Y., Shen H., Li J., Xia Y., Cheng Z. (2022). Circular RNA ACTN4 promotes intrahepatic cholangiocarcinoma progression by recruiting YBX1 to initiate FZD7 transcription. J. Hepatol..

[39] Wang X., Xing L., Yang R., Chen H., Wang M., Jiang R., Zhang L., Chen J. (2021). The circACTN4 interacts with FUBP1 to promote tumorigenesis and progression of breast cancer by regulating the expression of proto-oncogene MYC. Mol. Cancer.

[40] Gu Y., Wang Y., He L., Zhang J., Zhu X., Liu N., Wang J., Lu T., He L., Tian Y. (2021). Circular RNA circIPO11 drives self-renewal of liver cancer initiating cells via Hedgehog signaling. Mol. Cancer.

[41] Yang L., Han B., Zhang Z., Wang S., Bai Y., Zhang Y., Tang Y., Du L., Xu L., Wu F. (2020). Extracellular Vesicle-Mediated Delivery of Circular RNA SCMH1 Promotes Functional Recovery in Rodent and Nonhuman Primate Ischemic Stroke Models. Circulation.

[42] Panda A.C., De S., Grammatikakis I., Munk R., Yang X., Piao Y., Dudekula D.B., Abdelmohsen K., Gorospe M. (2017). High-purity circular RNA isolation method (RPAD) reveals vast collection of intronic circRNAs. Nucleic Acids Res..

[43] Pandey P.R., Rout P.K., Das A., Gorospe M., Panda A.C. (2018). RPAD (RNase R treatment, polyadenylation, and poly(A)+ RNA depletion) method to isolate highly pure circular RNA. Methods.

[44] Zhang X.-O., Dong R., Zhang Y., Zhang J.-L., Luo Z., Zhang J., Chen L.-L., Yang L. (2016). Diverse alternative back-splicing and alternative splicing landscape of circular RNAs. Genome Res..

[45] Westholm J.O., Miura P., Olson S., Shenker S., Joseph B., Sanfilippo P., Celniker S.E., Graveley B.R., Lai E.C. (2014). Genome-wide analysis of drosophila circular RNAs reveals their structural and sequence properties and age-dependent neural accumulation. Cell Rep..

[46] Gao Y., Wang J., Zhao F. (2015). CIRI: An efficient and unbiased algorithm for de novo circular RNA identification. Genome Biol..

[47] Wang K., Singh D., Zeng Z., Coleman S.J., Huang Y., Savich G.L., He X., Mieczkowski P., Grimm S.A., Perou C.M. (2010). MapSplice: Accurate mapping of RNA-seq reads for splice junction discovery. Nucleic. Acids Res..

[48] Hansen T.B. (2018). Improved circRNA identification by combining prediction algorithms. Front. Cell Dev. Biol..

[49] Rabin A., Zaffagni M., Ashwal-Fluss R., Patop I.L., Jajoo A., Shenzis S., Carmel L., Kadener S. (2021). SRCP: A comprehensive pipeline for accurate annotation and quantification of circRNAs. Genome Biol..

[50] Hardwick S.A., Chen W.Y., Wong T., Deveson I.W., Blackburn J., Andersen S.B., Nielsen L.K., Mattick J.S., Mercer T.R. (2016). Spliced synthetic genes as internal controls in RNA sequencing experiments. Nat. Methods.

[51] Chen I., Chen C.Y., Chuang T.J. (2015). Biogenesis, identification, and function of exonic circular RNAs. Wiley Interdiscip. Rev. RNA.

[52] Xu W., Seok J., Mindrinos M.N., Schweitzer A.C., Jiang H., Wilhelmy J., Clark T.A., Kapur K., Xing Y., Faham M. (2011). Inflammation and Host Response to Injury Large-Scale Collaborative Research, P. Human transcriptome array for high-throughput clinical studies. Proc. Natl. Acad. Sci. USA.

[53] Robinson M.D., McCarthy D.J., Smyth G.K. (2010). edgeR: A Bioconductor package for differential expression analysis of digital gene expression data. Bioinformatics.

[54] McCarthy D.J., Chen Y., Smyth G.K. (2012). Differential expression analysis of multifactor RNA-Seq experiments with respect to biological variation. Nucleic. Acids Res..

[55] Chen Y., Lun A.T., Smyth G.K. (2016). From reads to genes to pathways: Differential expression analysis of RNA-Seq experiments using Rsubread and the edgeR quasi-likelihood pipeline. F1000Research.

